# Vasodilatory Effect of Guanxinning Tablet on Rabbit Thoracic Aorta is Modulated by Both Endothelium-Dependent and -Independent Mechanism

**DOI:** 10.3389/fphar.2021.754527

**Published:** 2021-12-01

**Authors:** Yun Ling, Jiajun Shi, Quanxin Ma, Qinqin Yang, Yili Rong, Jiangmin He, Minli Chen

**Affiliations:** ^1^ Animal Experimental Research Center, Academy of Traditional Chinese Medicine, Zhejiang Chinese Medical University, Hangzhou, China; ^2^ Zhejiang Academy of Traditional Chinese Medicine, Hangzhou, China; ^3^ The Department of Medicine, Chiatai Qingchunbao Pharmaceutical Co., Ltd., Hangzhou, China; ^4^ Laboratory of Integrative Chinese and Western Medicine for the Diagnosis and Treatment of Circulatory Diseases of Zhejiang Province, Hangzhou, China

**Keywords:** vasodilation, calcium channel, endothelial NO synthase, calmodulin-dependent protein kinase II (CaMKII), Guanxinning tablet

## Abstract

Vasodilatory therapy plays an important role in the treatment of cardiovascular diseases, especially hypertension and coronary heart disease. Previous research found that Guanxinning tablet (GXNT), a traditional Chinese compound preparation composed of *Salvia miltiorrhiza* (Danshen) and *Ligusticum chuanxiong* (Chuanxiong), increase blood flow in the arteries, but whether vasodilation plays a role in this effect remains unclear. Here, we found that GXNT significantly alleviated the vasoconstriction of isolated rabbit thoracic aorta induced by phenylephrine (PE), norepinephrine (NE), and KCl in a dose-dependent manner with or without endothelial cells (ECs). Changes in calcium ion levels in vascular smooth muscle cells (VSMCs) showed that both intracellular calcium release and extracellular calcium influx through receptor-dependent calcium channel (ROC) declined with GXNT treatment. Experiments to examine potassium channels suggested that endothelium-denuded vessels were also regulated by calcium-activated potassium channels (K_ca_) and ATP-related potassium channels (K_ATP_) but not voltage-gated potassium channels (k_v_) and inward rectifying potassium channels (K_IR_). For endothelium-intact vessels, the nitric oxide (NO) and cyclic guanosine monophosphate (cGMP) contents in vascular tissue obviously increased after GXNT treatment, and pretreatment with the NO synthase inhibitor Nw-nitro-L-arginine methyl ester (L-NAME) or guanylyl cyclase inhibitor methylthionine chloride (MB) significantly inhibited vasodilation. An assessment of NO-related pathway protein expression revealed that GXNT enhanced the expression of phosphorylated endothelial NO synthase (eNOS) in a dose-dependent manner but had no effect on total eNOS, p-Akt, Akt, or PI3K levels in human umbilical vein ECs (HUVECs). In addition to PI3K/AKT signaling, Ca^2+^/calmodulin (CaM)-Ca^2+^/CaM-dependent protein kinase II (CaMKII) signaling is a major signal transduction pathway involved in eNOS activation in ECs. Further results showed that free calcium ion levels were decreased in HUVECs with GXNT treatment, accompanied by an increase in p-CaMKII expression, implying an increase in the Ca^2+^/CaM-Ca^2+^/CaMKII cascade. Taken together, these findings suggest that the GXNT may have exerted their vasodilative effect by activating the endothelial CaMKII/eNOS signaling pathway in endothelium-intact rings and calcium-related ion channels in endothelium-denuded vessels.

## Introduction

Blood vessels are an important part of the cardiovascular system. Targeted regulation of vasoconstriction or vasodilation is one of the most commonly used methods for the treatment of cardiocerebrovascular diseases, especially acute ischemia in conditions such as angina and stroke. In fact, as a treatment, vasodilators are widely used in dealing with many common diseases, including hypertension, coronary heart disease, preeclampsia, and chronic kidney disease (Croteau et al., 2014; Howlett, 2014).

Mature blood vessels are mainly composed of two parts: vascular smooth muscle cells (VSMCs) in the outer layer, whose contraction and relaxation directly determine the contraction and relaxation of blood vessels, and endothelial cells (ECs) in the inner layer, which secrete regulatory factors to regulate vasoconstriction and relaxation. Both of them determine the contraction or relaxation of blood vessels. Therefore, the regulation of vascular contraction and relaxation can be divided into endothelium-dependent and -independent mechanisms based on whether ECs are involved (Knox et al., 2019).

It is now believed that vasoconstriction is directly caused by the increase in intracellular calcium concentration, which arises from two processes: the release of endogenous calcium from the endoplasmic reticulum or mitochondria and the influx of extracellular calcium via potential-dependent channel (PDC) and receptor-dependent calcium channel (ROC). PDC is open when an action potential is produced, which is mainly caused by high-concentration ion stimulation or other ion channel openings. ROC is open when VSMCs are stimulated by various mediators or hormones, such as phenylephrine (PE) and norepinephrine (NE), through adrenergic and other receptors (Triggle, 1983). When the intracellular Ca^2+^ increases, Ca^2+^ further binds with calmodulin (CaM) to form a Ca^2+^/CaM complex, which activates ATPase and initiates contraction.

Nitric oxide (NO), a mediator secreted from ECs, plays an important role in vasoconstriction regulation by binding with soluble guanylate cyclase (sGC) in VSMCs (Zhao et al., 2015). Activated sGC can transform guanosine triphosphate (GTP) into cyclic guanosine monophosphate (cGMP), which improves the utilization rate of Ca^2+^ and reduces the level of intracellular Ca^2+^, leading to the inhibition of Ca^2+^-mediated vasoconstriction (Evora et al., 2012). The production of NO in the vasculature is mainly regulated by endothelial NO synthase (eNOS), which is regulated by serine/threonine kinases. PI3K/AKT signaling is a signal transduction pathways with serine and threonine kinase activities that has been demonstrated to be closely related to eNOS in the vasculature under many physiological and pathological conditions (Lin et al., 2018). Phosphorylation of AKT at Ser473 increases phosphorylation of the downstream effector eNOS at Ser1177, which activates eNOS and increases the release of NO from ECs (Walther et al., 2014). In ECs, Ca^2+^/CaM-Ca^2+^/calmodulin-dependent protein kinase II (CaMKII) signaling is another major signal transduction pathway for eNOS activation. As a multimeric serine/threonine kinase, CaMKII is initially activated by binding to Ca^2+^/CaM (Li et al., 2016). The activation of CaMK II has been found to promote the phosphorylation of eNOS and increase the release of NO (Murthy et al., 2017).

Guanxinning (GXN) is a classic Chinese herbal formula preparation extracted from *Salvia miltiorrhiza* (Danshen) and *Ligusticum chuanxiong* (Chuanxiong) at a ratio of 1:1. Guanxinning tablet (GXNT), as a powdered GXN extract, has been used for the clinical treatment of stable angina pectoris in coronary heart disease in China. It is proven that GXNT exerts antioxidant and antithrombotic effects in myocardial ischemia and coronary artery stenosis (Li et al., 2012; Li et al., 2020), but the mechanism has not been clearly elucidated. Clinical and animal studies have revealed that GXNT can effectively improve ischemia and increase blood flow in coronary arteries and cerebral vessels (Ying et al., 2005; Sun et al., 2019). Vasodilatory regulation is an important physiological response to improve blood flow. However, whether it is involved in the ability of GXNT to improve infarction and ischemia is still unknown. Our previous studies have shown that GXNT significantly increased the plasma level of NO in rats with myocardial ischemia–reperfusion injury (Hu et al., 2017). In this study, we investigated the effect of GXNT on the vasodilation of isolated rabbit aortic rings and further studied its mechanism.

## Materials and Methods

### Materials and Regents

GXNT (powdered GXN extract, crude herb content: 12.85 g/g) were provided by Chiatai Qinchunbao Pharmaceutical Co., Ltd. (Hangzhou, China). PE (No.7161001) and NE (No. 90302) were produced by Shanghai Hefeng Pharmaceutical Co., Ltd. (Shanghai, China). The inhibitors Nw-nitro-L-arginine methyl ester (L-NAME), methylthionine chloride (MB), 4-aminopyridine (4-AP), barium chloride (BaCl_2_), glibenclamide (GLI), tetraethylamine (TEA), and wortmannin were purchased from Sigma (United States). Metoprolol tartrate was obtained from AstraZeneca Pharmaceutical Co., Ltd. (UK), and diltiazem hydrochloride (Dil) injection was from Takata Pharmaceutical Co., Ltd. (Japan). Kits to detect NO (No.20180302) and cGMP (No. 20180208) were provided by the Nanjing Jiancheng Bioengineering Research Institute (Nanjing, China). The calcium ion indicator Fluo-4AM (KR652) was obtained from Japan DOJINDO Chemical Technology Co., Ltd (Japan). Primary antibodies against p-eNOS (9571S), eNOS (5880S), p-Akt (9271S), Akt (4691S), β-actin (4970S), p-CaMKII (12716S), and PI3K (4249S) were purchased from Cell Signaling Technology, Inc. (United States). The composition of Krebs solution, NaCl, KCl, KH_2_PO_4_, MgSO_4_, NaHCO_3_, CaCl_2_, and glucose, were purchased from Sigma.

### Preparation and Composition Analysis of Guanxinning Tablet

#### Preparation of Guanxinning Powder

Extraction of GXN powder was conducted as previously described (Wang et al., 2020). In brief, *Salvia miltiorrhiza* and *Ligusticum chuanxiong* (10 kg each) were added to 160 L of water, which was heated and kept at a low boil for 2 h. After pouring out the supernatant, the remaining residue was extracted twice again with 120 L of water and boiled for 1.5 h. All the supernatants were combined and concentrated to approximately 9 L at 60°C in a rotary evaporator, followed by the addition of 35 L of a 95% ethanol solution and overnight incubation. The supernatant was concentrated and dried completely at 60°C before being ground into a uniform powder by a grinder. The solid powder weighed approximately 1.5 kg (crude herb content 12.85 g/g).

#### Chemical Analysis of Powdered Guanxinning Components

The chemical composition of the GXN powder was analyzed with a high-performance liquid chromatography (HPLC) system (LC-20A, Shimadzu, Kyoto, Japan) and mass spectrometer (MS) detector system (API 3200, Agilent, California, United States) according to a previous protocol (Wang et al., 2020). GXN powder (1.0 g) was weighed and dissolved in a 50% methanol solution to 20 ml. The suspension was ultrasonicated for 10 min and then filtered through a 0.45-μm microporous filter column for HPLC-MS analysis. Five microliters of the prepared sample was injected with the mobile phase, which consisted of acetonitrile and 0.1% formic acid, and the following gradient program was run: 0–5 min, 0–40% A; 5–25 min, 40–69% A; 25–30 min, 69–100%. Data were collected and processed with Analyst software (version 1.6). *Salvia miltiorrhiza*, *Ligusticum chuanxiong*, and the monomer samples of each component were compared under the same conditions. A total of 14 major active components were isolated and identified in GXNT as in the description of Wang et al. (2020).

### Animals

Twenty-six New Zealand (NZ) white rabbits [Conventional animal (CV) grade, weight: 2.0–2.5 kg, half male and female] were provided by the Xinjian Rabbit Farm [license SCXK (Zhejiang) 2015–0004] in Dashi Town, Xinchang County, and raised in the Animal Experiment Research Center of Zhejiang Chinese Medicine University, under an environmental temperature of (21 ± 1)°C, a relative humidity of 40%–70%, and a noise level of <60 dB. They were housed in single cages and kept on a 12/12-h light/dark cycle with enough water and chow. Ethical approval for the present study was obtained from the Institutional Animal Care and Use Committee at Zhejiang Chinese Medicine University.

### Preparation of Isolated Thoracic Aortic Rings

Isolated thoracic aortic rings were prepared according to our previous research (Shou et al., 2012). Briefly, after the NZ white rabbits had been sacrificed by air embolism, the thoracic aorta was immediately removed without clots and adhesive connective tissues. Then it was cut into about 3- to 4-mm-long ring segments. Endothelium-denuded aortic rings were prepared with a mechanical method using cotton pipe cleaners. The absence of endothelium was confirmed by the absence of relaxation in response to 3 mM acetylcholine. Two hooks were used to keep the isolated aortic rings in a 5-ml organ bath containing Krebs–Henseleit (K-H) solution continuously aerated with a gaseous mixture of 95% O_2_ and 5% CO_2_ and maintained at 37°C. One hook was mounted at the bottom of the organ bath, and the other was connected via a micrometric manipulator to a force displacement transducer for the measurement of isometric force. Changes in isometric tension were recorded with a MedLab Biological Signal Collection System (medease Science and Technology, Nanjing, China). All preparations were allowed to equilibrate for 60 min before starting the experiment and the K-H solution was changed every 15 min.

The composition of the K-H solution includes the following: 118 mM NaCl, 4.7 mM KCl, 25 mM NaHCO_3_, 1.2 mM KH_2_PO_4_, 2.5 mM MgSO_4_,2.5 mM CaCl_2_, and 11.1 mM glucose. Except for CaCl_2_, the calcium-free K-H solution has the same composition as the K-H solution.

### Measurement of the Vascular Response to Guanxinning Tablet With or Without Phenylephrine, Norepinephrine, or KCl

After equilibration in K-H solution for 2 h, the aortic rings were successively incubated with GXNT at a series of concentrations (0.25, 0.5, 1, 2, 4, and 8 mg/ml) by cumulative concentration. The concentration of GXNT increased every 8 min, and changes in tension were recorded. When aortic rings were precontracted, PE, NE, and KCl at final concentrations of 10^−6^, 10^−6^, and 6 × 10^−2^ M, respectively, were added to the perfusate 20 min before the GXNT was added. The control group was treated with normal saline instead of the GXNT. The maximal contraction amplitude induced by PE, NE, and KCl was regarded as 100% contraction, and changes in vascular tension were calculated as follows:

Change in vascular tension (%) = amplitude of vascular tension/maximal contraction amplitude × 100%.

### Assessment of the Roles of Both Intracellular Ca^2+^ and Extracellular Ca^2+^

Aortic rings were equilibrated in K-H solution for 2 h before they were incubated with K-H solution containing 10^−6^ M PE. After 10 min of contraction, the specimen was washed with calcium-free K-H solution until the contraction tension restored to the basic tension value. PE (10^−6^ M) was added again after 30 min of equilibration. The rapid contraction of vascular ring was caused by the release of intracellular Ca^2+^. Changes in tension caused by intracellular Ca^2+^ were recorded. When tension was up to maximum, CaCl_2_ was added until 2.5 mM. The changes in tension, which stand for the changes caused by extracellular Ca^2+^, were recorded. The total tension change caused by intracellular Ca^2+^ and extracellular Ca^2+^ was regarded as 100% contraction, and changes in vascular tension were calculated as follows:

Change in vascular tension (%) = amplitude of vascular tension/total contraction amplitude × 100%.

For the GXNT treatment group, the rings were washed with the calcium-free K-H solution and then treated with 1.0, 2.0, and 4.0 mg/ml GXNT for 15 min. The above steps were repeated, and the results were compared.

### Assessment of Vasodilatory Inhibition by Nw-Nitro-L-Arginine Methyl Ester, Methylthionine Chloride, Metoprolol, Diltiazem Hydrochloride, Glibenclamide, Barium Chloride, Tetraethylamine, 4-Aminopyridine, and Wortmannin

The vascular rings were balanced in K-H solution for 2 h. PE (10^−6^ M) was added until the tension was stable, and then GXNT at final concentrations of 0.5, 1, 2, 4, and 8 mg/ml was added to the bath every 8 min. The change in tension was recorded, and then vascular rings were flushed with K-H solution to restore the tension to the previous equilibrium level. Inhibitors including the NO synthase inhibitor L-NAME (10^−4^ M), the guanylate cyclase inhibitor MB (10^−5^ M), the β receptor inhibitor metoprolol (10^−6^ M), the L-type Ca^2+^ channel inhibitor Dil (10^−5^ M), the ATP-sensitive K^+^ channel (K_ATP_) inhibitor GLI (10^−5^ M), the inward rectifier K^+^ channel (K_IR_) inhibitor BaCl_2_ (10^−3^ M), the Ca^2+^-dependent K^+^ channel (K_Ca_) inhibitor TEA (10^−3^ M), the voltage-gated K^+^ channel (K_v_) inhibitor 4-AP (10^−3^ M), or the PI3K inhibitor wortmannin (10^−7^ M) were added to the bath. After 30 min of incubation, the rings were incubated with PE and GXNT again, and the tension changes were recorded. The differences in tension values before and after the addition of inhibitors were compared.

Tension changes were recorded throughout the process. The control group was treated with normal saline instead of the GXNT. The maximal contraction amplitude induced by PE was regarded as 100% contraction, and changes in vascular tension were calculated as follows:

Change in vascular tension (%) = amplitude of vascular tension/maximal contraction amplitude × 100%.

### Cell Culture and Viability Determination

Human umbilical vein ECs (HUVECs) were purchased from Shanghai Cell Bank of Chinese Academy of Sciences and cultured in RPMI-1640 medium containing 10% neonatal bovine serum, 100 IU/ml of penicillin, and 100 μg/ml of streptomycin. They were placed in an incubator with 5% CO_2_ saturation at 37°C. Cell viability of HUVECs was measured by tetramethylazozolium salt (MTT) assay. In brief, cells were seeded in 96-well plates at a density of 1 × 10^5^ cells/ml and placed in the incubator containing 5% CO_2_ for 24 h. GXNT at a series of final concentrations (0.25, 0.5, 1, 1.5, 2, 3, 4, and 5 mg/ml) was added into plates and kept in the incubator for another 24 h. After 50 μl of 2 mg/ml MTT was added and incubated for 4 h, the absorbance of the wells was measured at 490 nm with an optical density (OD) value. The cell viability was calculated with the following formula:

Cell survival rate (%) = OD value of drug-treated cells/OD value of control cells × 100%.

### Detection of the Nitric Oxide and Cyclic Guanosine Monophosphate Contents

The rings were balanced and precontracted in K-H solution containing 10^−6^ M PE for 20 min. After 45 min of incubation with 1, 2, or 4 mg/ml of GXNT, the rings were immediately transferred to a −80°C freezer. Before the measurement, blood vessels from each group were weighed (0.5 g) and homogenized in a tissue homogenizer with 1 ml of ice-cold physiological saline. The supernatant was diluted 10 times with precooled PBS and centrifuged at 3,000 rpm for 10 min. The NO and cGMP contents in the vascular tissue were determined with kits according to the instructions of the manufacturers, and the tissue protein content was determined by the Coomassie brilliant blue method.

For HUVECs, after incubation with 0.25, 0.5, and 1 mg/ml GXNT for 24 h, the supernatant was collected and centrifuged at 2,000 rpm for 5 min before the NO content was determined.

### Determination of Calcium Concentration in Endothelial Cells

The calcium concentration was determined with a commercial kit according to the instructions of the manufacturer. Briefly, HUVECs were trypsinized and collected in a 50-ml centrifuge tube. They were washed twice with HBSS and centrifuged at 1,500 rpm for 5 min. Then, 2 ml of a working solution of Fluo-4AM, a calcium fluorescent probe, was added to the cell precipitation (ensuring that the liquid completely covered the cells), which was gently mixed and incubated at 37°C in the dark for 30 min. Then the cells were washed with HBSS three times, and the fluorescence value was detected in a black 96-well plate. After the addition of 0.25, 0.5, or 1 mg/ml of GXNT, the fluorescence value was detected every 10 min. The instantaneous and continuous changes in fluorescence values were plotted, respectively. The change rate was calculated as T_N_/T_0_ × 100%, where T_N_ represents the fluorescence value at N min.

### Western Blotting

The total proteins of HUVECs were extracted according to the instructions of commercial assay kits (Jiangsu KeyGEN BioTECH Co. Ltd, Nanjing, China). After quantification by the bicinchoninic acid (BCA) protein assay, cytosolic proteins were mixed with a 5× loading buffer and heated in a boiling water bath for 10 min. Then equal amounts of proteins were separated by 8%–12% SDS-PAGE and transferred to polyvinylidene difluoride (PVDF) membranes (Millipore, Bedford, MA, United States). After blocking with 5% nonfat milk in TBS for 2 h at room temperature, the membranes were incubated with primary antibodies (p-eNOS: 1:500 dilution, eNOS: 1:800 dilution, p-Akt: 1:500 dilution, Akt: 1:800 dilution, β-Actin: 1:1,000 dilution, p-CaMK II: 1:500 dilution, and PI3K: 1:800 dilution) at 4°C overnight followed by IRDye™800 conjugated secondary antibody for 2 h at room temperature. Visualization and quantization were performed with an Odyssey Infrared Imaging System (LI-COR Inc., Lincoln, NE, United States). All the cytosolic protein bands were normalized to the corresponding β-actin content.

### Statistical Analysis

All data were presented as mean ± S.D. and SPSS 19.0 statistical software was used for data analysis. The least significant difference method (LSD) test of one-way ANOVA was used for comparisons between different groups. Differences were considered statistically significant when *p* < 0.05.

## Results

### The Vasodilatory Effect of Guanxinning Tablet on Isolated Rabbit Thoracic Aortic Rings is Partially Dependent on the Intact Endothelium

**FIGURE 1:**
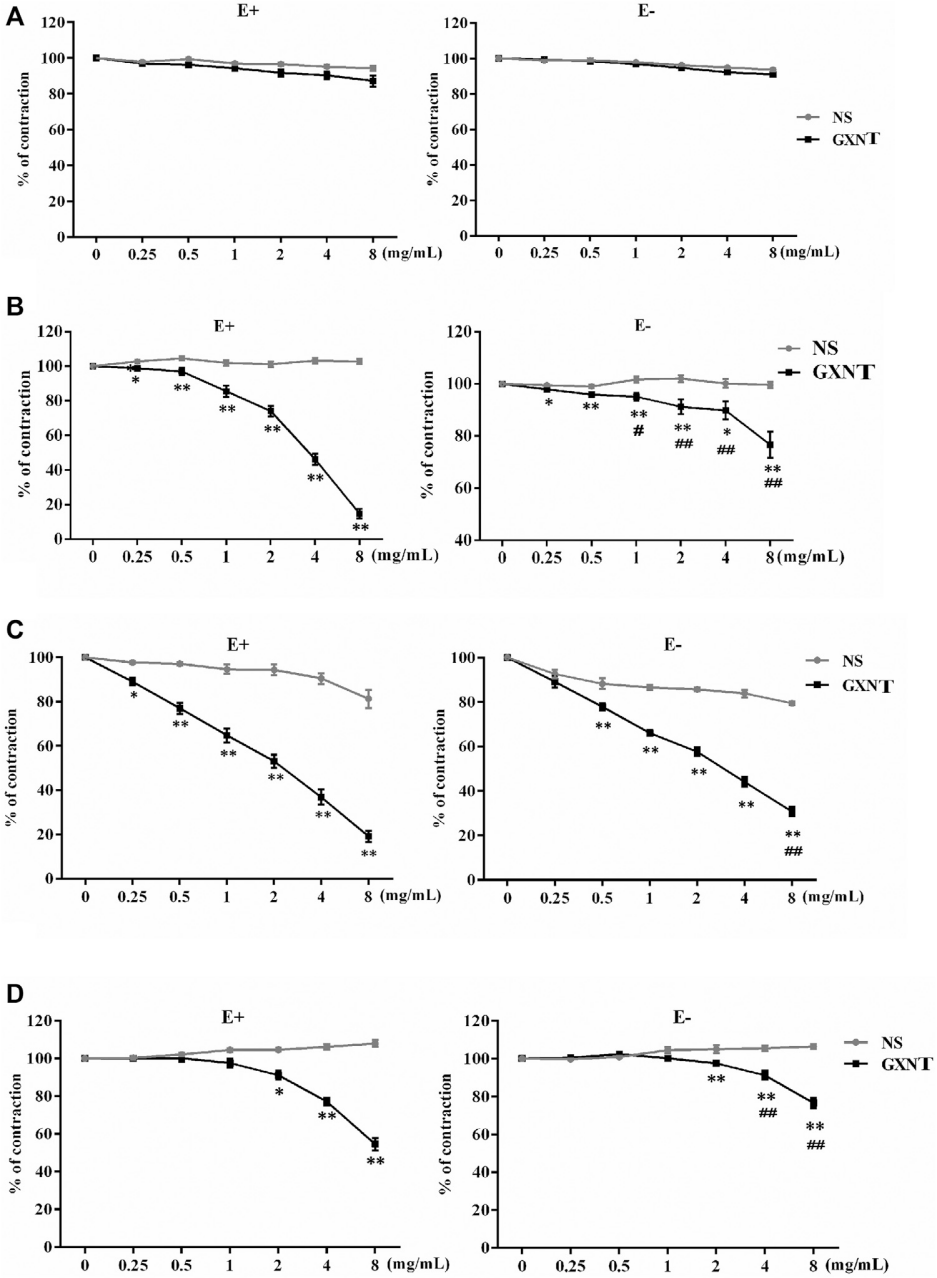
Effects of Guanxinning tablet (GXNT) on the contraction of isolated thoracic aortic rings induced by phenylephrine (PE), norepinephrine (NE), and KCl. After equilibration in Krebs–Henseleit (K–H) solution, vascular rings with or without endothelium were pretreated with normal saline **(A)**, 10^−6^ mol/L PE **(B)**, 10^−6^ mol/L NE **(C)**, or 60 mmol/L KCl **(D)** for 20 min. Then, GXNT was added to the solution to 0.25, 0.5, 1, 2, 4, and 8 mg/ml. The concentration of GXNT was increased every 8 min, and changes in tension were recorded after stabilization. The vasoconstrictive tension before addition of the drug was taken as 100% of the normal control group, and the tension after addition of the drug action was compared with it and is reported as a percentage. (*p < 0.05, **p < 0.01 compared with the NS group, ^*##*^ *p* < 0.01 compared with vascular rings with intact endothelium). Results are presented as the mean ± SD of the data obtained in three independent experiments with six duplicate samples.

The isolated rabbit thoracic aorta model is a classic model used to investigate the effect of drugs on vasoconstriction. As shown in Figure 1A, 0–8 mg/ml of GXNT had no significant effect on the tension of the vascular ring in the basal state with or without ECs. After administration of GXNT at final concentrations of 0.25–8 mg/ml, the vascular rings with intact endothelium precontracted with 10^−6^ mol/L PE, 10^−6^ mol/L NE, or 60 mmol/L KCl were significantly relaxed in a dose-dependent manner (Figures 1B–D). To further clarify whether the inhibitory effect of GXNT on vasoconstriction is related to ECs, endothelium-denuded rings were also tested in the same way. With increasing GXNT dose, the remission of precontracted aortic rings clearly increased (Figures 1B–D). However, compared with vascular rings with intact endothelium, the degree of relief in aortic rings precontracted by 10^–6^ mol/L PE or 60 mmol/L KCl was decreased after GXNT treatment, which suggested that ECs may participate in the vasodilatory effect of GXNT.

### Guanxinning Tablet Plays a Vasodilating Role in Endothelium-Denuded Rings by Affecting Calcium and Potassium Channels, Rather Than *β* Receptor

**FIGURE 2:**
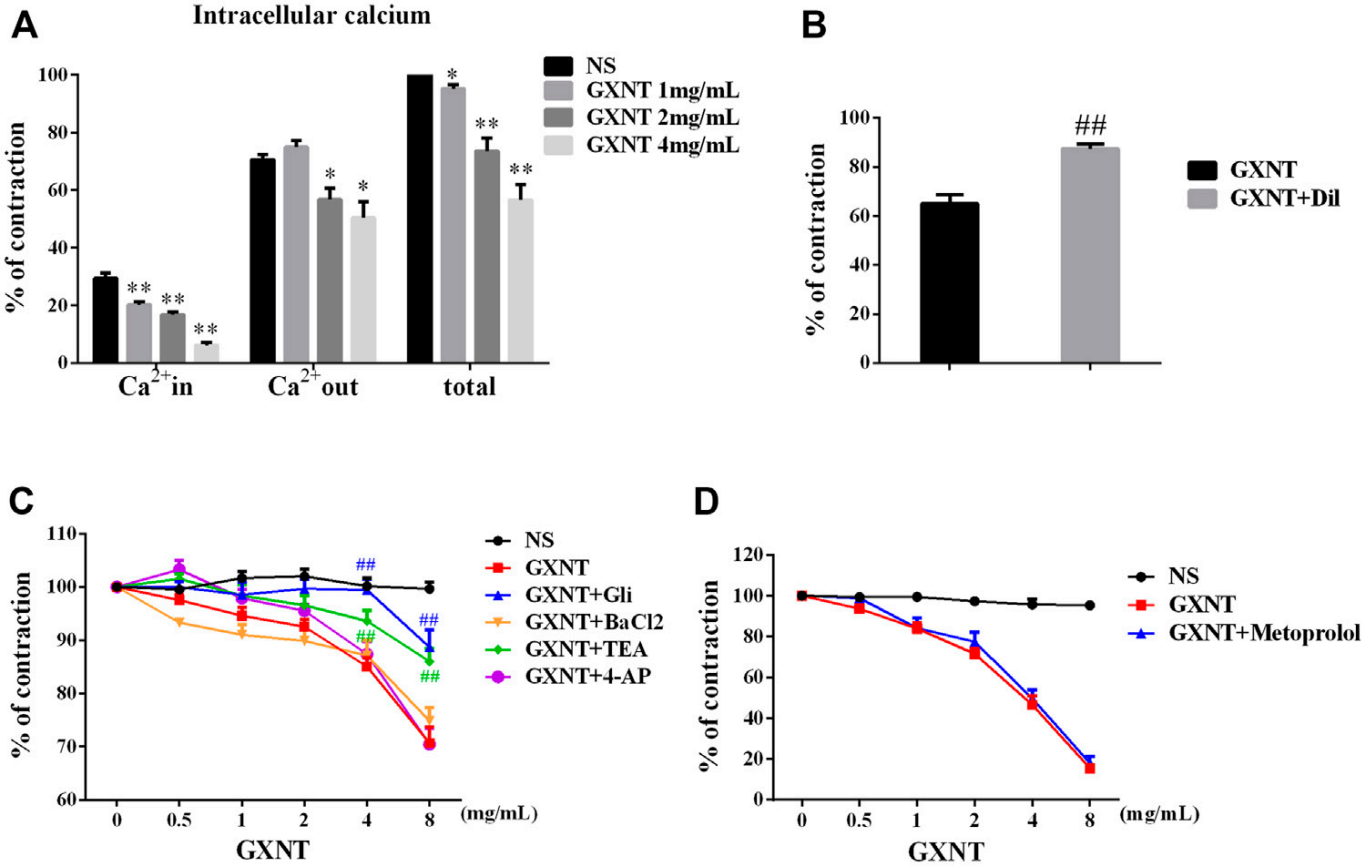
Effects of GXNT on ion channels and β receptors in endothelium-denuded aortic rings. **(A)** Changes in intracellular and extracellular calcium in endothelium-denuded aortic rings. **(B)** Influences of calcium influx on the vasodilatory effect of GXNT. Diltiazem hydrochloride (Dil) (10^−5^ M), a calcium influx blocker, was given 30 min before 10^–6^ mol/L PE, and 4 mg/ml of GXNT was added, and the tension was recorded and compared. **(C)** Effects of different potassium channels on the vasodilation effect of GXNT. Rings were preincubated with the K_Ca_ inhibitor tetraethylamine (TEA) at 10^−3^ M, K_ATP_ inhibitor glibenclamide (GLI) at 10^−5^ M, the K_v_ inhibitor 4-aminopyridine (4-AP) at 10^−3^ M, and the K_IR_ inhibitor barium chloride (BaCl_2_) at 10^−3^ M for 30 min followed by the addition of 10^−6^ mol/L of PE and GXNT at increasing concentrations of 0.5, 1, 2, 4, and 8 mg/ml. The differences in tension before and after addition of the inhibitors were compared. **(D)** The effect of β receptor blockade on the vasodilatory effect of GXNT was tested. Metoprolol (10^−6^ M) was applied as pretreatment 30 min before 10^−6^ mol/L PE and GXNT were added in turn. In the NS group, PE and GXNT were not given, but normal saline was used instead (*p < 0.05, **p < 0.01 compared with the NS group, ^*##*^ *p* < 0.01 compared with the GXNT group). The vasoconstrictive tension before the addition of the drug was taken as 100% of the normal control group, and the tension after the addition of the drug was compared with it and is reported as a percentage. Results are presented as the mean ± S.D. of the data obtained in three independent experiments with six duplicate samples.

Since ECs and VSMCs can affect vascular dilation, respectively, we first investigated the effect of GXNT on endothelium-denuded vessels. A change in the intracellular calcium concentration is key to the contraction or relaxation of VSMCs. We observed that GXNT significantly reduced the degree of contraction during calcium-free perfusion and the ability to undergo continuous contraction after the addition of a calcium solution to endothelium-denuded rings, which implies that it affects both internal and external calcium (Figure 2A). Pretreatment with Dil, an L-type Ca^2+^ channel inhibitor, for 20 min significantly reduced the vasodilatory effect of 8 mg/ml of GXNT (Figure 2B). As mentioned, when potassium channels open, calcium influx decreases, and VSMCs relax. Thus, we further examined the influences of GXNT on different potassium channels of VSMCs. In this experiment, we found that the K_Ca_ inhibitor TEA and K_ATP_ inhibitor GLI could inhibit the vasodilatory effect of GXNT, while the K_v_ inhibitor 4-AP and the K_IR_ inhibitor BaCl_2_ had no significant effect on the vasodilatory effect of GXNT. These results suggest that K_Ca_ and K_ATP_ are involved in the vasodilatory effect of GXNT, while K_v_ and K_IR_ are not (Figure 2C). Among the adrenergic receptors, α receptors regulate vasoconstriction, and β receptors regulate vasodilation. Because GXNT affected vasoconstriction, the influence of GXNT on β receptors on VSMCs was also tested. After administration of metoprolol, a β-receptor inhibitor, no significant difference in endothelium-denuded rings precontracted by PE with and without GXNT treatment was noted (Figure 2D).

### Guanxinning Tablet Exerts Endothelium-Mediated Vasodilation by Promoting Nitric Oxide Production to Activate the Nitric Oxide/Soluble Guanylate Cyclase/Cyclic Guanosine Monophosphate Pathway

**FIGURE 3:**
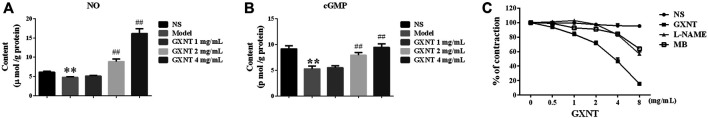
GXNT promoted nitric oxide (NO) production in endothelium-intact vascular rings and further activated the soluble guanylate cyclase (sGC)–cyclic guanosine monophosphate (cGMP) expression. NO **(A)** and cGMP **(B)** contents in endothelium-intact vascular rings after GXNT treatment. After precontraction with 10^−6^ M PE, rings with intact endothelium were incubated with 1, 2, and 4 mg/ml GXNT for 45 min and immediately collected for homogenization. Before testing with an ELISA kit, homogenate supernatants were centrifuged at 3,000 rpm for 10 min. The model indicates treatment with PE without GXNT, NS indicates treatment with only normal saline. **(C)** Impact of GXNT on NO synthesis and GC in endothelium-intact vascular rings. Aortic rings were preincubated with the NOS synthase inhibitor Nw-nitro-L-arginine methyl ester (L-NAME) (10^−4^ M) and guanylate cyclase inhibitor methylthionine chloride (MB) (10^−5^ M) for 30 min. After pretreatment with 10^−6^ M PE, the rings were bathed in GXNT for 30 min. The change in tension was noted and quantified by the ratio of tension after GXNT and inhibitor administration to the contraction tension with PE. (**p < 0.01 compared with the NS group, ^*##*^ *p* < 0.01 compared with the model group). Results are presented as the mean ± SD of the data obtained in three independent experiments with six duplicate samples.

ECs secrete NO and activate the NO/sGC/cGMP pathway, which is a common regulatory mechanism for endothelium-dependent vasodilation. To elucidate the vasodilator mechanism of GXNT, we investigated the influence of GXNT on NO and NO signaling. The NO and cGMP contents in vascular homogenate with PE-mediated precontraction with or without GXNT treatment were tested (Figures 3A,B). After incubation with GXNT at 2 and 4 mg/ml for 45 min, the NO and cGMP contents in the vascular rings were significantly increased. To further confirm the mechanism of vasodilation by GXNT through NO, NO synthase inhibitor L-NAME and guanylate cyclase inhibitor MB were used, respectively. The vasodilation of vascular rings induced by GXNT after PE precontraction was significantly inhibited w *i*th L-NAME and MB preincubation (Figure 3C).

### Guanxinning Tablet Activated the Phosphorylation of Endothelial Nitric Oxide Synthase in Human Umbilical Vein Endothelial Cells *Via* PI3K/AKT-Independent Mechanisms

**FIGURE 4:**
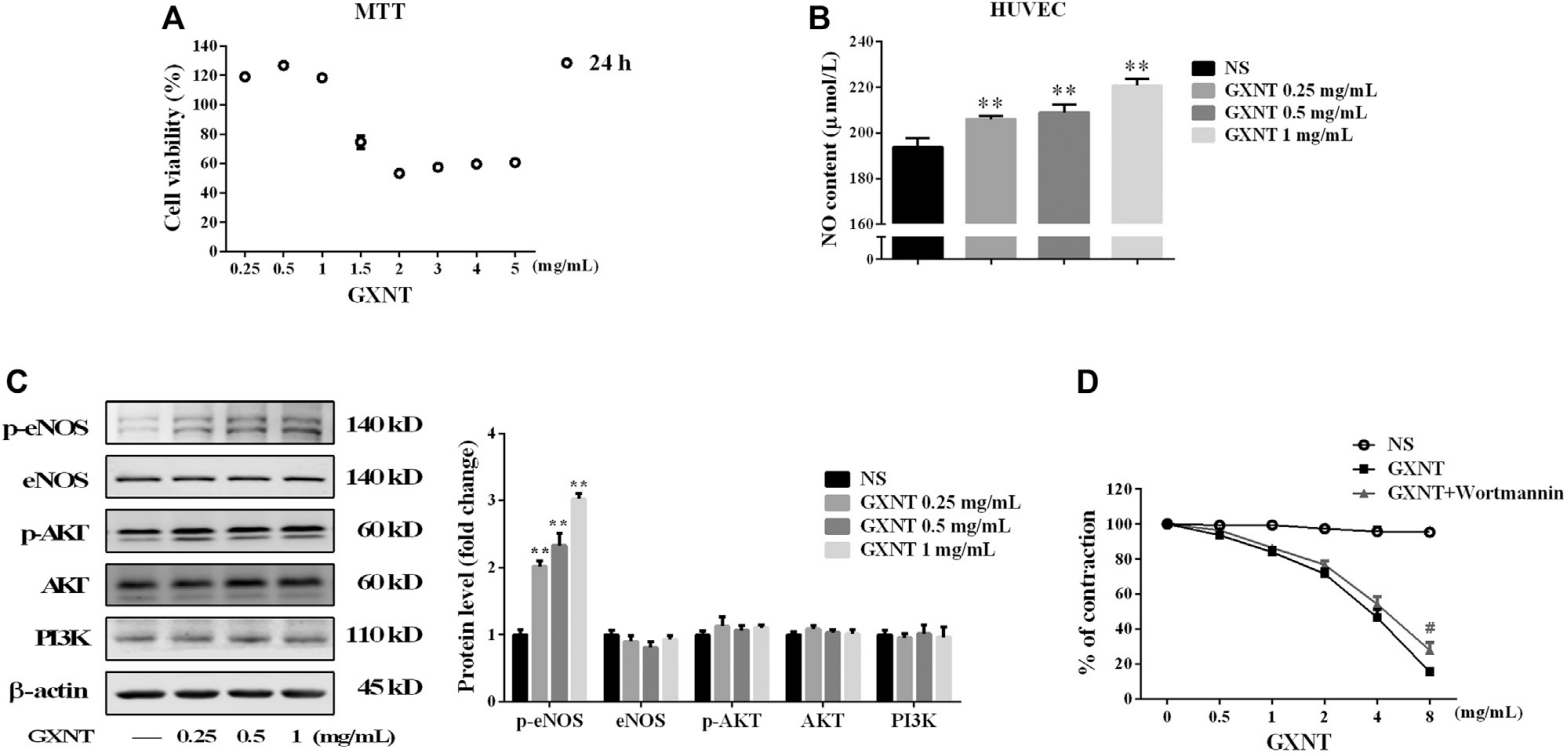
Effects of GXNT on NO synthesis and the related endothelial nitric oxide synthase (eNOS)/PI3K/AKT signaling pathway. **(A)** Cell viability of human umbilical vein endothelial cells (HUVECs) treated with GXNT at a series of final concentrations (0.25, 0.5, 1, 1.5, 2, 3, 4, and 5 mg/ml) for 24 h. **(B)** NO content in the cell supernatant. HUVECs were treated with 0.25, 0.5, and 1 mg/ml GXNT for 24 h. Then the cell supernatants were collected and centrifuged at 2,000 rpm for 5 min before the levels of NO were determined by ELISA with a kit. **(C)** Influence of GXNT on the eNOS/PI3K/AKT-mediated NO-related signaling pathway in HUVECs. Cells were incubated with 0.25, 0.5, and 1 mg/ml GXNT for 24 h, and then the proteins were extracted and determined by Western blotting. Proteins were quantified according to the gray value of the bands, and the fold changes represent the ratio of other groups to the NS group. **(D)** Effect of a PI3K inhibitor on the vasodilatory effect of GXNT. Endothelium-intact rings (*n* = 6) were preincubated with wortmannin (10^−7^ M) for 30 min before precontracted with 10^−6^ M PE. After treatment with GXNT, the change in tension was quantified as the ratio of tension after GXNT and inhibitor administration to the contraction tension with PE (**p < 0.01 compared with the NS group, ^*#*^ *p* < 0.05 compared with the GXNT group). Results are presented as the mean ± SD of data obtained in three independent experiments.

To further elucidate the mechanism by which GXNT enhances NO production, HUVECs were used. As shown in Figures 4A,B, GXNT at or below 1 mg/ml had no significant inhibitory effect on HUVEC viability, and GXNT significantly increased the level of NO in the cell culture supernatant. Considering the effect of NOS synthase inhibitor on vasodilatory effect of GXNT in aortic rings, we examined the protein expression of total eNOS and p-eNOS in HUVECs treated with GXNT. A dose-dependent raise was observed in the levels of p-eNOS at Ser 1,177 after treatment with 0.25, 0.5, and 1 mg/ml of GXNT, while no significant change was seen in the total protein expression of eNOS (Figure 4C). The PI3K/AKT signal pathway is an important upstream target of eNOS, and phosphorylation of Akt at serine 473 (Ser473) can increase the phosphorylation of eNOS at Ser1177. Thus, we explored protein expression of total AKT and p-AKT at Ser473 in GXNT-treated HUVECs. However, the protein levels of total AKT and p-AKT did not change after GXNT treatment as well as the expression of PI3K. Moreover, the PI3K inhibitor wortmannin did not significantly alleviate the vasodilatory effect of GXNT in endothelium-intact aortic rings, which suggested that GXNT may raise the activity of eNOS in a PI3K/AKT-independent manner (Figure 4D).

### Guanxinning Tablet Induces Calmodulin-Dependent Protein Kinase II Activation by Promoting Intracellular Free Calcium Binding

**FIGURE 5:**
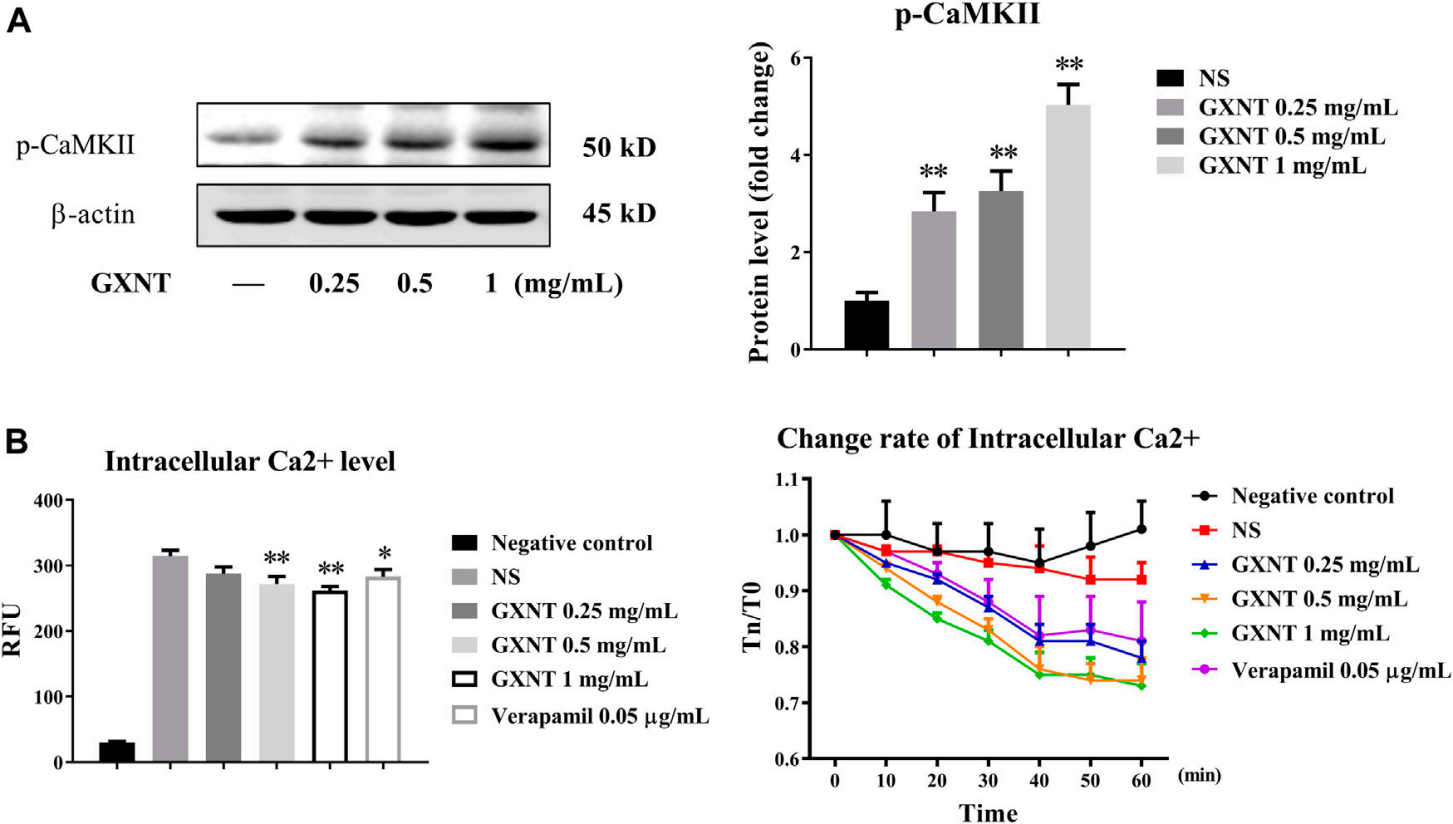
Activation of calmodulin-dependent protein kinase II (CaMKII) in endothelial cells (ECs) by GXNT. **(A)** Protein expression of p-CAMKII in HUVECs treated with GXNT. HUVECs were incubated with 0.25, 0.5, and 1 mg/ml GXNT for 24 h before protein levels were tested by Western blotting. Proteins were quantified according to the gray value of the bands, and the fold changes represent the ratio of other groups to the control group. **(B)** The effect of GXNT on intracellular free calcium of ECs. HUVECs preloaded with a fluorescent probe were treated with 0.25, 0.5, and 1 mg/ml of GXNT, and the change in Ca^2+^ was detected immediately (left, 0 min), and continuous change rate (right, 0–60 min) was also observed every 10 min. The change rate was calculated as T_N_/T_0_ × 100%, where T_N_ represents the fluorescence value at N min (*p < 0.05, **p < 0.01 compared with the NS group). Results are presented as the mean ± SD of data obtained in three independent experiments.

In addition to PI3K/AKT, CaMKII is another important serine/threonine kinase that regulates eNOS by transducing intracellular calcium (Koo et al., 2018). The results of protein expression analysis showed that the phosphorylation of CaMKII protein was remarkably increased by GXNT (Figure 5A). The binding of free calcium to CaM in ECs can activate Ca^2+^/CaMKII and promote the activation of eNOS. Thus, the change in calcium concentration in ECs can reflect the binding degree with CaM. Examination of the intracellular calcium level test showed that the free calcium level in HUVEC cytoplasm decreased significantly in a GXNT concentration-dependent manner (Figure 5B), which indicated that GXNT may promote more free Ca^2+^ binding to CaM and then activate CaMKII and eNOS.

## Discussion

Long-term hypertension can lead to many cardiovascular diseases and their complications, such as coronary heart disease, heart failure, stroke, renal fibrosis, and ocular vascular diseases and so on. Vasodilation is one of the main treatment strategies for hypertension (Maher and Dworkin, 1996; Luo et al., 2021). Although the vasodilators currently used in clinical practice are effective, their long-term use produced obvious side effects (Loh et al., 2020). GXNT contains a powder extracted from a classic Chinese herbal formula used for the treatment of coronary heart disease. Here, we found that GXNT significantly inhibited vasoconstriction of the isolated thoracic aorta induced by PE, NE, and KCl in both endothelium-dependent and -independent ways. Vasodilation was significantly influenced by ion channels, including internal calcium, external calcium, and potassium channels, but not the β receptor in endothelium-denuded rings, and vasodilation in endothelium-intact rings was promoted by activating the synthesis of NO and the NO–SGC–cGMP pathway. Mechanistic research in HUVECs showed that GXNT increased the phosphorylation of eNOS and synthesis of NO in a PI3K/AKT-independent manner through Ca^2+^/CaM/CaMKII activation.

Vascular resting tension is the result of the interaction between ECs and VSMCs, which balances blood vessel contraction and relaxation. We found that GXNT could relax the aortic ring induced by PE, NE, and KCl with or without endothelium, which illustrated its impact on both the endothelium and smooth muscle to exert its vasodilatory effect. However, differences in the effects of these compounds exist. PE and NE mainly cause vasoconstriction by activating the α receptor (Chen and Shepherd, 1991); thus, the contraction intensity is significantly higher than that inducted by KCl. Moreover, compared with the PE group, the NE group displayed decreased dependence on the endothelium; thus, PE was used to induce contraction in the next endothelial experiment.

Considering that GXNT caused vasodilation of the rings regardless of the presence of endothelium, we further studied the mechanism of the vasodilatory effect of GXNT on rings with or without endothelium. When the endothelium is removed, contraction of the rings mainly depends on the smooth muscle itself. The contraction of blood vessels regulated by many factors and changes in the calcium ion concentration are their common main basis (Gurney and Clapp, 1994). We found that GXNT significantly increased internal calcium release and external calcium influx in VSMCs. K^+^ channels play an important role in membrane potential. The opening of K^+^ channels on VSMC membrane can promote K^+^ outflow, lead to hyperpolarization of membrane potential, the closing of PDC on the cell membrane, a reduction in calcium influx and relaxation of the vascular smooth muscle (Boz et al., 2016). After the use of Ca^2+^ and K^+^ channel inhibitors, respectively, the diastolic effects were significantly inhibited by blocking Ca_L_, K_ca_, and K_ATP_, which indicated that GXNT may relax VSMCs by regulating Ca^2+^- and ATP-related signaling channels and that Ca^2+^ and K^+^ channels may equally contribute to the relaxation of VSMCs induced by GXNT. In addition, since the β-blocker could not inhibit the vasodilatory effect of GXNT at all, our results suggested that the opening of calcium and potassium channels may be the mechanism of the vasodilatory effect of GXNT on VSMCs.

NO is an important vasodilator, which plays the role through NO–SGC–cGMP pathway in VSMCs and mainly regulated by eNOS in endothelium (Dupont et al., 2014). eNOS is influenced by various stimuli, among which stress is the most common activator of the PI3K/Akt pathway in ECs (Xing et al., 2015). Thus, we examined the expression level of Akt, p-Akt, and PI3K in HUVECs, but they were not significantly changed after GXNT treatment. The calcium ion level in ECs also affects the activity of eNOS through CaMKII (Murthy et al., 2017). Our results demonstrated that the phosphorylation of CaMKII protein levels was remarkably increased, and the free calcium level in the EC cytoplasm was significantly decreased by GXNT. Considering that CaMKII does not depend on ion channels in the cell membrane, such as L-type Ca^2+^ and voltage-dependent K^+^ channels (Pellicena and Schulman, 2014), we did not examine intracellular calcium outflow, and the decrease in intracellular free calcium was attributed to an increase in calcium ion binding to calmodulin, which activates CaMKII.

So far, we have found 14 main components in the component identification of GXNT (Wang et al., 2020). Some of them may have contributions to the vasodilatory effect of GXNT. Among these components, phenylalanine (Mitchell et al., 2004), chlorogenic acid (Suzuki et al., 2006), ferulic acid (Choi et al., 2012), and salvianolic acid B (Shou et al., 2012) have been found to have endothelial NO-related vasodilatory effects, which is consistent with the results of GXNT found by us, but except salvianolic acid B, there is no in-depth study on the mechanism of others. The mechanism of salvianolic acid B is different from that of GXNT, suggesting that the effect of GXNT on CaMKII may come from other active ingredients. In terms of ion channels, there were many compounds with calcium or potassium ion channel activities in vessels, including phenylalanine (Tan et al., 2020), ferulic acid (Zhou et al., 2017), and salvianolic acid B (Lam et al., 2006), and more compounds had the activity in other cells, such as salvianolic acid A in cardiomyocytes (Bao, 1993), isosalvianolic acid C (Lin et al., 2019) in mast cells, and so on. In addition, compounds with calmodulin activity include phenylalanine (VanScyoc et al., 2002), chlorogenic acid (Tong et al., 2007), and salvianolic acid A (Qiang et al., 2015), but only chlorogenic acid was reported to be related to endothelial calmodulin. Thus, there is no compound whose activity and mechanism of action are completely consistent with GXNT, but most of our findings are supported by some reports of active ingredients, suggesting that the vasodilatory mechanism of GXNT may be the result of their joint action.

Many vasodilators have different dilation effects on large and small vessels, which can affect some specific diseases. For example, the dilation of large vessels, such as the coronary artery, can alleviate myocardial ischemia (Simsek et al., 2021), while the dilation of small vessels increases peripheral resistance (Jensen et al., 2019). At present, our research is carried out on only the rabbit thoracic aorta, which is a large vessel. The effects of GXNT on small vessels will be investigated in our follow-up research work.

In conclusion, as a potential vasodilator drug, GXNT exerted significant vasodilatory effects in rabbit thoracic aortic rings by opening calcium and potassium channels in VSMCs and activating the CaMKII/eNOS signaling pathway in vascular ECs. Our results add a new mechanism to explain the cardiovascular protection of GXNT and provide an experimental basis for the clinical application of GXNT.

## Data Availability

The raw data supporting the conclusion of this article will be made available by the authors, without undue reservation.

## Abbreviations

4-AP, 4-aminopyridine; BaCl_2_, barium chloride; CaM, calmodulin-dependent protein; CaMKII, calmodulin-dependent protein kinase II; cGMP, cyclic guanosine monophosphate; Dil, diltiazem hydrochloride; eNOS, endothelial NO synthase; GLI, glibenclamide; GXNT, Guanxinning tablet; K-H solution, Krebs–Henseleit solution; K_Ca_, Ca2^+^-dependent K^+^ channel; K_ATP_, ATP-sensitive K^+^ channel; K_v_, voltage-gated K^+^ channel; K_IR_, inward rectifier K^+^ channel; L-NAME, Nw-nitro-L-arginine methyl ester; MB, methylthionine chloride; NE, norepinephrine; PDC, potential-dependent channel; PE, phenylephrine; ROC, receptor-dependent calcium channel; sGC, soluble guanylate cyclase; TEA, tetraethylamine; VSMCs, vascular smooth muscle cells.
